# Modified Charlson Comorbidity Index to Improve Management of Patients with Hepatocellular Carcinoma: A Step Towards Multiparametric Approach

**DOI:** 10.3390/cancers18071151

**Published:** 2026-04-02

**Authors:** Eleonora Alimenti, Massimo Iavarone, Elia Fracas, Lorenzo Canova, Mariangela Bruccoleri, Barbara Antonelli, Anna Maria Ierardi, Pierpaolo Biondetti, Angelo Sangiovanni, Cristiano Quintini, Gianpaolo Carrafiello, Pietro Lampertico

**Affiliations:** 1 Division of Gastroenterology and Hepatology, Foundation IRCCS Ca’ Granda Ospedale Maggiore Policlinico, 20122 Milan, Italy; eleonora.alimenti@policlinico.mi.it (E.A.); mariangela.bruccoleri@policlinico.mi.it (M.B.); angelo.sangiovanni@policlinico.mi.it (A.S.); pietro.lampertico@unimi.it (P.L.); 2 CRC “A. M. and A. Migliavacca” Center for Liver Disease, Department of Pathophysiology and Transplantation, University of Milan, 20122 Milan, Italy; 3 Department of Pathophysiology and Transplantation, Università degli Studi di Milano, 20122 Milan, Italy; elia.fracas@unimi.it; 4 Division of Hepatology, San Giuseppe Hospital, IRCCS MultiMedica, 20122 Milan, Italy; lorenzo.canova@multimedica.it; 5 General and Liver Transplant Surgery Unit, Foundation IRCCS Ca’ Granda Ospedale Maggiore Policlinico, 20122 Milan, Italy; barbara.antonelli@policlinico.mi.it (B.A.); cristiano.quintini@unimi.it (C.Q.); 6 Department of Diagnostic and Interventional Radiology, Foundation IRCCS Ca’ Granda Ospedale Maggiore Policlinico, 20122 Milan, Italy; annamaria.ierardi@policlinico.mi.it (A.M.I.); gianpaolo.carrafiello@unimi.it (G.C.); 7 Radiology Department, Foundation IRCCS Ca’ Granda Ospedale Maggiore Policlinico, 20122 Milan, Italy; pierpaolo.biondetti@policlinico.mi.it

**Keywords:** HCC, systemic therapy, ablation, surgery, liver transplantation

## Abstract

**Background and aims:** Hepatocellular carcinoma (HCC) patients frequently present with comorbidities that limit therapeutic options and increase mortality. This study evaluated the performance of the Charlson Comorbidity Index (CCI) and a modified CCI (mCCI) in stratifying patients with HCC to predict treatment allocation and survival. **Methods:** A retrospective single-center cohort study analyzed 401 patients with de novo HCC (74% male, median age 68 years, 80% Child–Pugh-Turcotte (CPT) A, 65% viral etiology, 70% Barcelona Clinic Liver Cancer stage (BCLC) 0/A). CCI and mCCI (with points related to HCC and chronic liver disease excluded), were calculated at diagnosis for each patient. The primary endpoint was overall survival (OS) estimated by Kaplan–Meier method and compared across mCCI classes; Cox uni/multivariable models were applied to identify predictors of mortality. The secondary aim was evaluating the association between mCCI and treatment allocation. **Results:** While CCI classified 94% of patients as “high-risk”, mCCI reclassified patients into “high-risk” (21%), “intermediate-risk” (48%), and “low-risk” (31%), demonstrating better stratification whilst maintaining a strong correlation with CCI (Kendall’s tau-b = 0.57, *p* < 0.001). BCLC B patients with “high-risk” mCCI exhibited significantly lower access to first-line curative treatment (14% vs. 47%, *p* = 0.03). Moreover, “high” or “intermediate-risk” patients according to mCCI experienced significantly shorter OS compared to “low-risk” (median OS 36 vs. 49 vs. 74 months, *p* < 0.001). “High-risk” and “intermediate-risk” mCCI classes were independent predictors of mortality, alongside alpha-fetoprotein, CPT and BCLC stage. Considering the items composing mCCI, age and cardiovascular diseases were independent predictors of mortality. **Conclusions:** mCCI provides a more accurate assessment of comorbidities than the standard CCI and is associated with survival, hence it can contribute to designing patient-tailored therapeutic strategies.

## Simple Summary

This study proposes a modified Charlson Comorbidity Index (mCCI) that excludes points for liver disease and HCC itself. The mCCI is developed and validated to address the limitations of the standard Charlson Comorbidity Index (CCI) in the context of Hepatocellular Carcinoma (HCC). The mCCI offers a more refined assessment of the impact of extrahepatic comorbidities on treatment allocation and survival. The findings are of critical importance for the multidisciplinary teams involved in HCC management, as mCCI more accurately stratifies patients by comorbidity burden, particularly being associated with differences in access to curative treatments like resection or transplantation in intermediate-stage Barcelona Clinic Liver Cancer (BCLC)-B HCC. Teffihis is vital for avoiding undertreatment in patients with low comorbidity burden and overtreatment in those with significant extrahepatic conditions. In clinical practice, mCCI can serve as a pragmatic instrument to inform treatment decisions, particularly when assessing the risks and benefits of aggressive interventions in early-stage HCC.

## 1. Introduction

Hepatocellular carcinoma (HCC) is the most common primary liver cancer and a leading cause of morbidity and mortality worldwide [1]. Its development is often linked to pre-existing liver conditions, including cirrhosis, typically caused by viral infections, excessive alcohol consumption, and non-alcoholic fatty liver disease [2,3].

Staging of hepatocellular carcinoma is crucial for determining the most appropriate therapeutic approach, primarily relying on systems like the Barcelona Clinic Liver Cancer (BCLC) classification [4]. These frameworks consider factors such as the size and number of tumor lesions, liver function, and the presence of tumor-related symptoms, but age and comorbidities (such as cardiovascular disease and diabetes) are unaccounted for. However, these factors can significantly influence prognosis of patients with HCC and are often considered in treatment selection in clinical practice, potentially limiting available options.

A new multiparametric therapeutic hierarchy for managing patients with HCC has recently been proposed [5]. In this proposal, treatments are prioritized based on their proven survival benefit, irrespective of stage, allowing flexibility in choosing the most effective feasible treatment for the individual patient. However, the major novelty of this system is the multidisciplinary team assessment of several factors (patient frailty, comorbidities, tumor location, technical feasibility) to personalize treatment selection [5]. In particular, the proposal introduces the concept of ‘fit’ or ‘unfit’ for a given treatment. However, this assessment is left to the clinician in the absence of comprehensive studies for patients with HCC. The determination of the patient’s state of ‘fit/unfit’ can be influenced by various features, including the patient’s age, the underlying chronic liver disease and functional liver reserve, the presence of sarcopenia and frailty, and the existence of comorbidities. These factors may be present in the same patient with varying degrees of severity, and their simultaneous presence can influence the patient’s survival and the feasibility of the selected treatment modality. Therefore, the potential limitation of the “multiparametric therapeutic hierarchy” proposal pertains to the absence of clearly defined and quantifiable parameters to support the assessment of ‘fit/unfit’. This ambiguity can be attributed to a paucity of significant literature on the subject, which hinders its applicability and verifiability across diverse contexts. Among the various factors to be considered, the patient’s age and the presence of comorbidities could be assessed using the Charlson Comorbidity Index (CCI), which is a widely used scoring system for predicting one-year mortality in hospitalized patients and takes into account different comorbidities, each assigned a specific weight (points) based on their impact on one-year mortality [6]. Several studies have incorporated the CCI to assess the impact of comorbidities on outcomes in patients with chronic liver diseases, often in conjunction with other prognostic scores specific to liver disease (e.g., Child–Pugh score, model for end-stage liver disease (MELD) score). These studies often investigate the relationship between CCI score, liver-specific scores, and overall mortality or transplant-related outcomes [7,8,9,10]. However, even if studies have shown the prognostic significance of age and comorbidity in other cancers [11,12,13,14,15], evidence of the role of CCI in patients with HCC is scarce [16,17,18,19].

To summarize, several tools are available to assess prognosis in patients with HCC, however most of them focus primarily on tumor burden and liver function. Scores such as Child–Pugh–Turcotte, MELD, and ALBI capture the severity of underlying liver disease but do not account for extrahepatic comorbidity burden. Conversely, general frailty indices are not routinely used and often lack validation in patients with cirrhosis or HCC. Even if the Charlson Comorbidity Index (CCI) is widely used to quantify comorbidities, its application in HCC is problematic because the score includes both chronic liver disease and solid tumors (including HCC itself) as weighted variables, potentially inflating the comorbidity burden in this population. Therefore, adapting the CCI to better isolate extrahepatic comorbidities may provide a more clinically meaningful tool to support multidisciplinary treatment decision-making in patients with HCC. Herein, we evaluated the association between age and comorbidity burden included in the CCI with curative treatment receipt and overall survival (OS) in a large cohort of patients with first diagnosis of HCC.

## 2. Patients and Methods

This is a single center retrospective study including all patients with a first diagnosis of HCC managed at the Center for Liver Disease of Fondazione IRCCS Cà Granda Ospedale Maggiore Policlinico (Milan) between 1 January 2011 and 1 November 2022. Patients were longitudinally followed; clinical data were collected for the analysis along time and retrospectively revised. Inclusion criteria were first diagnosis of HCC, according to updated international guidelines (either histologically proven or by radiological criteria), and availability of complete clinical baseline information (at first diagnosis of HCC). For the particular purpose of the present analysis, other inclusion criterion was availability of information about comorbidity burden (i.e., pre-calculated CCI or single items: cardiological, pulmonary, vascular, neurological diseases, other tumors different from HCC, diabetes, connective tissue diseases, HIV infection with AIDS-defining complications, chronic kidney disease, peptic ulcer). Exclusion criteria were missing data about clinical outcomes in terms of survival during a follow-up of at least 3 months after HCC diagnosis. All procedures followed the “Strengthening the Reporting of Observational Studies in Epidemiology guideline” [20], and they complied with the ethical standards and with Helsinki Declaration of 1975, as revised in 2008. The study was approved by the Ethical Committee “Lombardia 3” (ID 4428_S_P, 24 January 2024). Informed consent was waived owing to the retrospective nature of the study, as was ensured by Italian law.

### 2.1. Study Endpoints

The primary endpoint of the study was to evaluate the association between comorbidity burden and OS, focusing on the relationship between CCI, a modified version tailored for cirrhotic patients (mCCI), and HCC prognosis. The secondary objectives were to evaluate whether treatment allocation and access to curative treatment observed in clinical practice differed across CCI or mCCI classes; to assess the association between CCI, mCCI and individual comorbidities with post-treatment survival in predefined clinical subgroups, as exploratory analyses.

### 2.2. HCC Diagnosis, Staging, Treatment Allocation and Response Evaluation

HCC was diagnosed by radiology [either by contrast enhanced computed tomography (CT)-scan or magnetic resonance (MR)] or by histology, according to updated international guidelines [21,22,23,24,25]. HCC was staged according to the BCLC staging system, including the following features: Child–Pugh–Turcotte score (CPT) [26], performance status by Eastern Cooperative Oncology Group (ECOG) [27], number and diameter of tumors, macrovascular invasion (MVI), and extrahepatic spread (EHS) [3,4,28,29]. According to the BCLC system, HCCs at first diagnosis were staged into very early (BCLC 0), early (BCLC-A), intermediate (BCLC B), advanced (BCLC C), and end-stage (BCLC-D). At the time of the first diagnosis of HCC (baseline), all patients underwent a CT scan of the chest, bone scintigraphy and upper gastrointestinal endoscopy (UEG), if not recently performed; these examinations were repeated whenever clinically relevant during follow-up.

First-line treatment allocation was based on the updated guidelines at the time of diagnosis [3,4,21,22,23,24,25,28,29]: each case was discussed by the multidisciplinary team (MDT) and allocated to treatment according to the up-to-date recommendations, recording in a specific report the reason why the decision deviated from the expected indications by disease stage (as recommended by guidelines), such as the presence of comorbidities, the availability of a certain type of treatment or other factors determining the decision. Treatments considered curative were liver transplantation (LT), surgical resection, radiofrequency/microwave thermal ablation (TA) and combination treatments (resection + TA or TA + transarterial chemoembolization (TACE), performed in the same therapeutic session). If a patient had received locoregional treatment (LRT) during the transplant waiting list (bridging treatments), LT was considered the up-front allocated treatment. Radiological response to treatment was assessed by either contrast enhanced CT-scan or MR performed (1) one month apart after LT and then every six months; (2) one month apart for LRTs or surgery (if complete response detected, then every 3–4 months in the first 2 years or until recurrence); (3) every other month for systemic treatment until progression, discontinuation of therapy for any reason or transition to supportive therapy only.

### 2.3. Definition of Etiology and Staging of Underlying Chronic Liver Disease

Liver disease etiology was classified as viral if patients had HCV infection (detectable HCV RNA) and/or chronic HBV infection (HBsAg or detectable HBV DNA), with or without HDV coinfection. All patients with viral infections received antiviral therapy per national and international guidelines. Non-viral etiologies included alcoholic liver disease (ALD; alcohol consumption >20 g/day in females, >30 g/day in males), metabolic-associated steatotic liver disease (MASLD; steatosis with diabetes, overweight/obesity, or ≥2 metabolic risk factors) [30], autoimmune hepatitis [31], and primary biliary cholangitis [32]. Mixed etiology was assigned when viral and non-viral causes coexisted, while cryptogenic liver disease was diagnosed when all causes were excluded.

Cirrhosis was diagnosed histologically or non-invasively (liver stiffness > 15 kPa by elastography or indirect signs of advanced liver disease, such as thrombocytopenia [platelet count <150,000 × 10^9^/L], varices, or portal hypertension features on imaging) [33]. Cirrhosis severity was assessed using the CPT score, liver function with MELD and albumin-bilirubin grade (ALBI) scores [34,35], and clinically significant portal hypertension was defined per Baveno consensus criteria [36]. Baseline characteristics at HCC diagnosis included patients’ demographics, comorbidities, presence of esophageal or gastric varices at last available UEG, and laboratory variables.

### 2.4. Charlson Comorbidity Index Definition

The Charlson Comorbidity Index (CCI) was calculated for each patient at HCC diagnosis using only comorbidities already present at baseline. The assessment of comorbidities was based on the retrospective review of patients’ electronic medical records which are systematically recorded in routine clinical practice within structured electronic medical records. For some patients, CCI was already available, and for others it was calculated using the comorbidities defining the score: history of myocardial infarction, chronic heart failure, peripheral vascular disease, cerebrovascular accidents or transient ischemic attack, dementia, chronic pulmonary disease, connective tissue disease, peptic ulcer disease, liver disease, diabetes mellitus, hemiplegia, chronic kidney disease, solid tumor, leukemia, lymphoma, AIDS. The definition of each comorbidity and assigned points were based on the original paper and are reported in Table 1 [6]. The mCCI was derived by subtracting points allocated for liver disease (0 to 3 points according to severity) and HCC as a solid tumor (2 points for localized and 6 points for metastatic), to identify the extrahepatic disease burden, as already performed in patients with cirrhosis in other settings [8]. If a patient had a pre-existing (and still present) or concomitant diagnosis of another solid tumor, points for the neoplasm were added to the score. Patients were stratified in 3 different risk classes, defined a priori, based on the stepwise increase in 1-year mortality observed across Charlson score strata in the original development cohort [6]: low risk (CCI 0–1), intermediate risk (CCI 2–3), and high risk (CCI ≥ 4). The same cut-offs were applied for mCCI to maintain the clinical interpretability of the score and comparability with prior literature.

### 2.5. Statistical Analysis

Continuous variables were expressed as median and interquartile range (IQR) and compared through the Kruskal–Wallis test. Categorical variables were reported as the number (percentage) and compared by Fisher’s exact test or chi-square when appropriate. In further analyses missing data were handled with a complete-case approach. Death was reported as percentage. Correlation between CCI and mCCI was tested through Kendall’s Tau-b Rank Correlation. The follow-up time started from first HCC diagnosis until death or the last available access to hospital facilities. OS was defined as the time from HCC diagnosis to death, while post-treatment survival was defined as the time from first-line HCC treatment to death: patients still alive at the time of data cut-off were censored at the last follow-up. Kaplan–Meier analysis was used for unadjusted estimation of OS and post-treatment survival across the CCI and mCCI classes, and survival curves were compared through log-rank test, meanwhile adjusted analyses were performed using Cox proportional hazard models. Specifically, univariable Cox proportional hazard models were applied to evaluate the association between baseline variables and mortality, after assessing the proportional hazard assumption using Schoenfeld residuals. The covariates included in the analysis were age, gender, mCCI, etiology of underlying liver disease, presence of cirrhosis and its complications (ascites, esophageal/gastric varices and hepatic encephalopathy), laboratory parameters (albumin, bilirubin, INR, creatinine, platelet count < 150 × 10^9^/mL) either considered individually or summarized into composite scores (ALBI, MELD, CPT), alpha-fetoprotein (AFP, analyzed both as a continuous and dichotomous variable using 200 ng/mL as threshold) [24,25,37], tumor characteristics (size and number of HCC nodules, presence of extrahepatic spread and/or macrovascular invasion, BCLC stage, presence of HCC exceeding the “Milan criteria”). To avoid collinearity, variables reflecting overlapping clinical characteristics (particularly liver function variables: albumin, bilirubin, MELD, ALBI, and Child–Pugh–Turcotte score) were not simultaneously included in the multivariable models. Covariates were selected a priori based on clinical relevance and only variables significant in univariable analysis (*p*-value < 0.05) were included in multivariable models. Multivariable Cox models were used to estimate the adjusted associations between comorbidity burden and survival outcomes in a prognostic framework. Analyses of post-treatment survival were conditional on receipt of first-line treatment. Subgroup analyses according to the BCLC stage and treatment modality were conducted as exploratory analyses. Results were reported as hazard ratios (HRs) and 95% confidence intervals (CI). The discriminative ability of mCCI score and of the multivariable models was quantified by Uno’s C index, while calibration was assessed by comparing predicted survival probabilities with observed Kaplan–Meier estimates and through the Brier score when appropriate. Model performance measures should be interpreted as internally derived, given the absence of external validation. Treatment allocation across CCI and mCCI risk classes was compared using Fisher’s exact test or the chi-square test to characterize differences in the distribution of treatment strategies across strata of comorbidity burden, without implying causal effects on treatment decisions. Data management and analysis were performed using the STATA SE 14.0 (StataCorp, 4905 Lakeway Dr, College Station, TX, USA) and RStudio 2025.05.1 + 513 “Mariposa Orchid” Release.

## 3. Results

### 3.1. Baseline Characteristics of Patients

**Figure 1:**
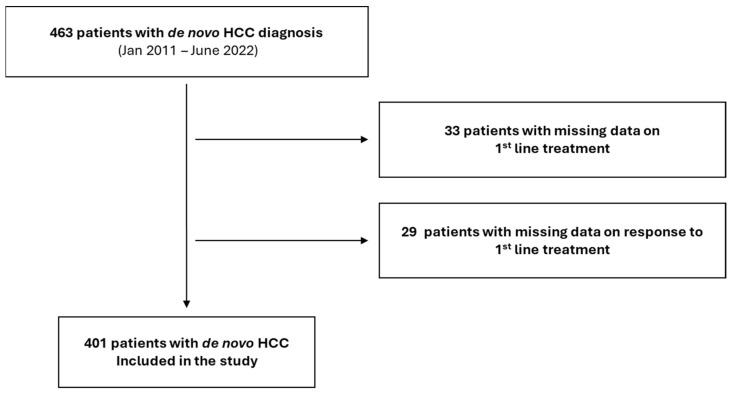
Patients’ disposition.

Between January 2011 and June 2022, 401 patients were diagnosed with de novo HCC in our Center and followed longitudinally until death or last visit (Figure 1).

Baseline characteristics of patients included in the study are reported in Table 2: 74% males, median age 68 years (IQR 60–75), the most frequent etiology of liver disease was HCV infection (55%), and 357 (89%) patients had a diagnosis of cirrhosis mainly with preserved liver function [CPT A in 309 (80%)]. HCC was staged BCLC 0 in 103 (26%) patients, A in 178 (44%), B in 67 (17%), C in 41 (10%) and D in 12 (3%). Among patients with BCLC D, 1 (8%) was considered for LT due to the tumor burden beneath “Milan criteria” and thus reclassified as BCLC A. Overall, HCC was a single nodule in 254 (63%) patients and median diameter of the largest nodule was 2.5 cm (IQR 1.7–4.1).

### 3.2. Patients’ Stratification at Baseline According to CCI and mCCI

**Figure 2:**
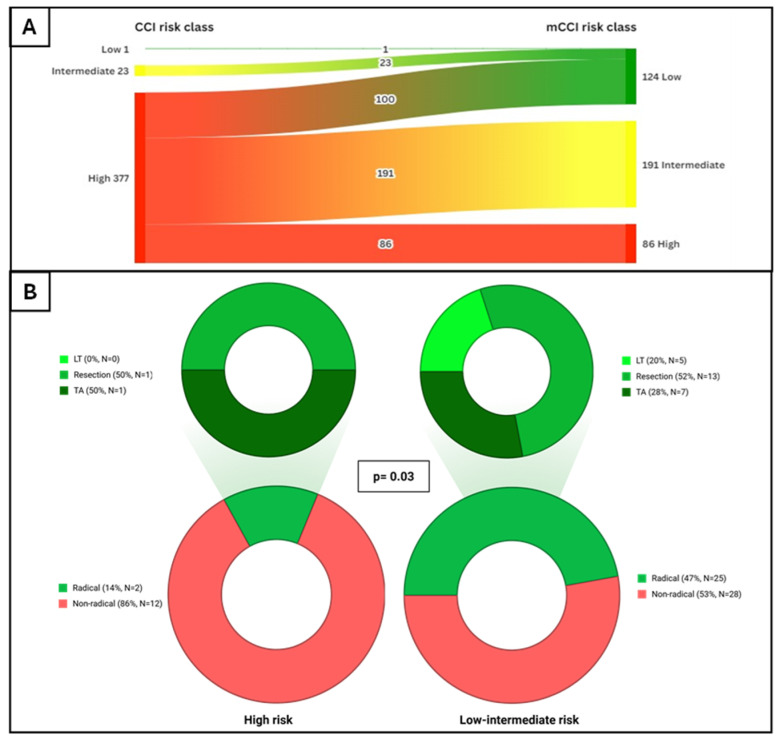
Patients’ distribution according to CCI and mCCI class. (**A**) Changes in classification of patients from Charlson Comorbidity Index (CCI) to modified Charlson Comorbidity index (mCCI); (**B**) Access to curative treatment in first line according to mCCI in the overall cohort.

CCI and mCCI showed a good correlation (Kendall’s tau-b = 0.57; *p*-value < 0.001). According to CCI, patients were stratified as 377 (94%) “high-risk”, 23 (5.75%) “intermediate-risk” and only 1 (0.25%) “low-risk”. After reclassifying patients according to mCCI, 86 (21%) were classified as “high-risk”, 191 (48%) as “intermediate-risk” and 124 (31%) as “low-risk” (Figure 2). The balanced distribution of patients across the three risk classes allowed meaningful comparisons of prognostic data. Due to the long period of enrollment, patients were divided in Cohort-1 (from 2011 to 2015, N = 233) and Cohort-2 (from 2016 to 2022, N = 168): the prevalence of mCCI risk classes was similar between the two groups (*p* = 0.45). The distribution of mCCI risk classes across BCLC stages is reported in Supplementary Table S1.

### 3.3. First-Line Treatment Allocation According to CCI and mCCI

**Figure 3:**
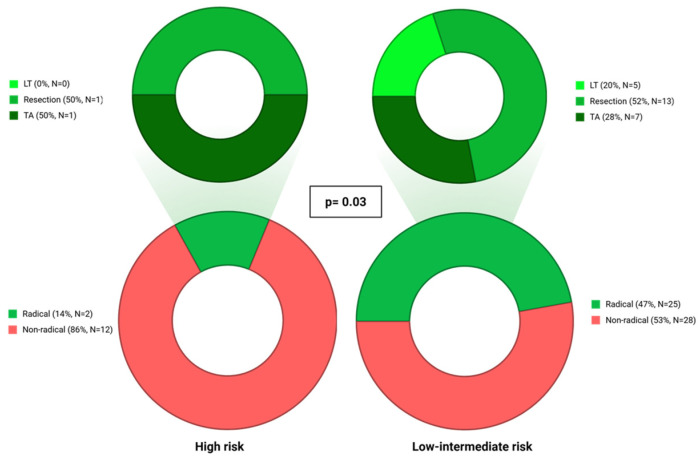
Access to curative treatment in first line according to mCCI in BCLC B.

The median time between HCC diagnosis and first-line treatment was 2 months (IQR 0.1–3). Overall, 239 (60%) patients received treatments with curative intent, 112 (28%) were treated with TACE, 4 (1%) with TARE, 33 (8%) with systemic therapy, and 13 (3%) patients received supportive care only. No significant differences were observed in treatment strategies across the three classes of CCI. Similarly, no significant differences in first-line treatment were observed overall and within each BCLC stage for mCCI classes (Supplementary Table S2). However, when patients with “low” and “intermediate-risk” mCCI are grouped together, a higher proportion of curative treatments compared with the “high-risk” was observed in BCLC B patients (N = 67, 47% vs. 14% *p*-value = 0.03; Figure 3). Moreover, among BCLC B patients receiving curative treatments, surgical approaches were less frequent among patients with high mCCI (50% vs. 72%, *p* = 0.07). This pattern became even more evident when limiting the analysis only to compensated BCLC B patients undergoing curative therapy, where the proportion undergoing surgery was significantly lower in “intermediate” or “high-risk” groups (low risk mCCI class 100% vs. intermediate 33% vs. high 50%, *p* = 0.03). Given the limited sample size, these subgroup analyses should be interpreted as exploratory.

### 3.4. Survival According to CCI and mCCI

During a median follow-up of 33 (IQR 16–68) months, 230 (57%) patients died: 49 (48%) BCLC 0, 93 (52%) BCLC A, 43 (64%) BCLC B, 34 (83%) BCLC C and 11 (92%) BCLC D. OS for the whole cohort was 52 months (IQR 25–94), with cumulative survival rates of 87.9% (95% CI 84.2–90.8) at 1, 75.3% (IQR 70.5–79.4) at 2 years, and of 49.3% (IQR 43.6–54.8) at 5 years.

Stratification according to CCI did not identify significant differences in OS between “high-risk” and “intermediate-risk” [52 (IQR 25–92) vs. 59 (IQR 49–109) months, *p* = 0.40]. Similar results were observed after dividing patients according to the year of HCC diagnosis in Cohort 1 (2011–2015, *p* = 0.24) and Cohort 2 (2016–2022, *p* = 0.45). The “low-risk” CCI class was not considered in the survival analysis as it included only one patient.

**Figure 4:**
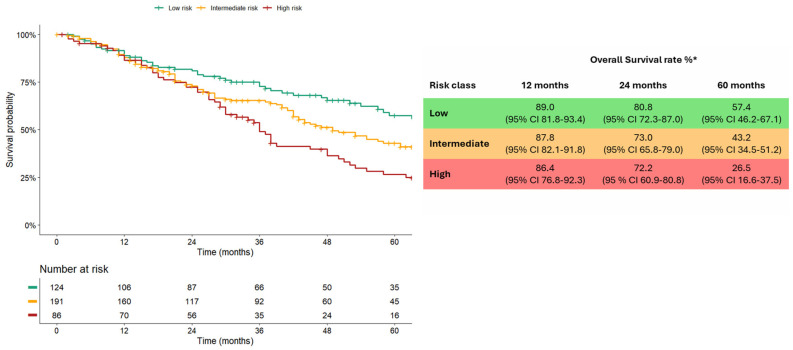
Overall 5-year survival rate stratified according to mCCI in the whole cohort. * cumulative survival rate.

In contrast, stratifying patients according to mCCI risk, median OS was significantly shorter in “high-risk” (36 [IQR 21–62] months) and “intermediate-risk” (49 [IQR 22–80] months) classes as compared to “low-risk” (74 [IQR 36–152] months, *p*-value < 0.001), Figure 4. Separation between the ‘low-risk’ and ‘intermediate/high-risk’ curves became evident after approximately 18 months of follow-up, whereas divergence between the ‘intermediate’ and ‘high-risk’ groups occurred later, around 30 months. Time dependent discrimination demonstrated that the prognostic contribution of comorbidities was variable over time. Discrimination of mCCI for mortality was limited in the short-term (mean AUC 0.52, 95% CI 0.44–0.60, at 1 year) but progressively increased during follow-up, reaching a mean AUC of 0.60 between 3 and 5 years (Supplementary Figure S1a). Similarly, calibration showed a good agreement between observed and predicted survival probabilities in the intermediate term (Supplementary Figure S1b).

In univariable Cox regression analysis, both “intermediate” and “high-risk” mCCI classes were associated with increased mortality as compared to the “low-risk” group (Table 3). The proportional hazard assumption tested with Schoenfeld residuals and cumulative hazard plots did not indicate significant violation (*p* = 0.23, Supplementary Figure S2). After multivariable adjustment for BCLC stage, AFP values, and liver function, “intermediate” and “high-risk” mCCI classes remained independently associated with mortality (HR 1.69, 95% CI 1.16–2.47, *p*-value 0.007; HR 2.80, 95% CI 1.81–4.33, *p*-value < 0.001; Table 3). The final multivariable model included established prognostic variables (BCLC stage, AFP values, CPT) together with mCCI risk classes. Inclusion of mCCI in the multivariable model provided prognostic information complementary to established predictors, as indicated by its independent association with mortality and the discriminative performance of the model. The good discriminative performance of the multivariable model was demonstrated by time-dependent AUC values of 0.80 (95% CI 0.72–0.87) at 1 year, 0.82 (95% CI 0.76–0.87) at 2 years, 0.79 (95% CI 0.73–0.85) at 3 years and 0.77 (95% CI 0.71–0.83), at 4 years (Supplementary Figure S3a). Consistently, calibration analysis showed good agreement between predicted and observed survival probabilities, with lower Brier scores compared with the null model across follow-up, indicating improved prediction accuracy. (Supplementary Figure S3b) These performance estimates represent internal validation within the study cohort as no formal optimism correction (e.g., bootstrap resampling) was performed.

The prognostic association between mCCI and survival was also confirmed after stratifying patients according to the year of HCC diagnosis, with higher comorbidity burden being consistently associated with shorter OS (in Cohort 1, 2011–2015: “high-risk” 38 [IQR 28–77] vs. “intermediate risk” 57 [IQR 25–87] vs. “low-risk” 74 [IQR 30–152] months, *p* = 0.002); in Cohort 2, 2016–2022: “high-risk” 30 (IQR 16–55) vs. “intermediate-risk” 43 (IQR 17–68) vs. “low-risk” NA (IQR 38-NA) months, *p*-value = 0.009).

**Figure 5:**
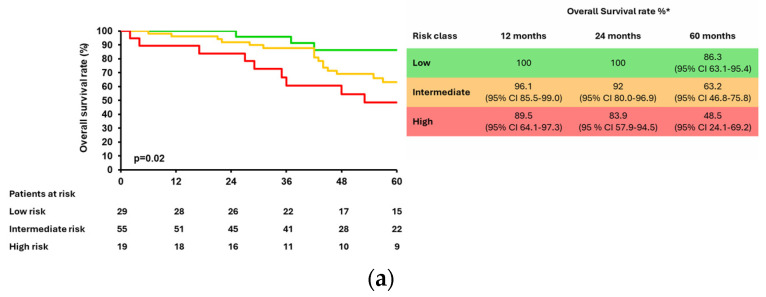

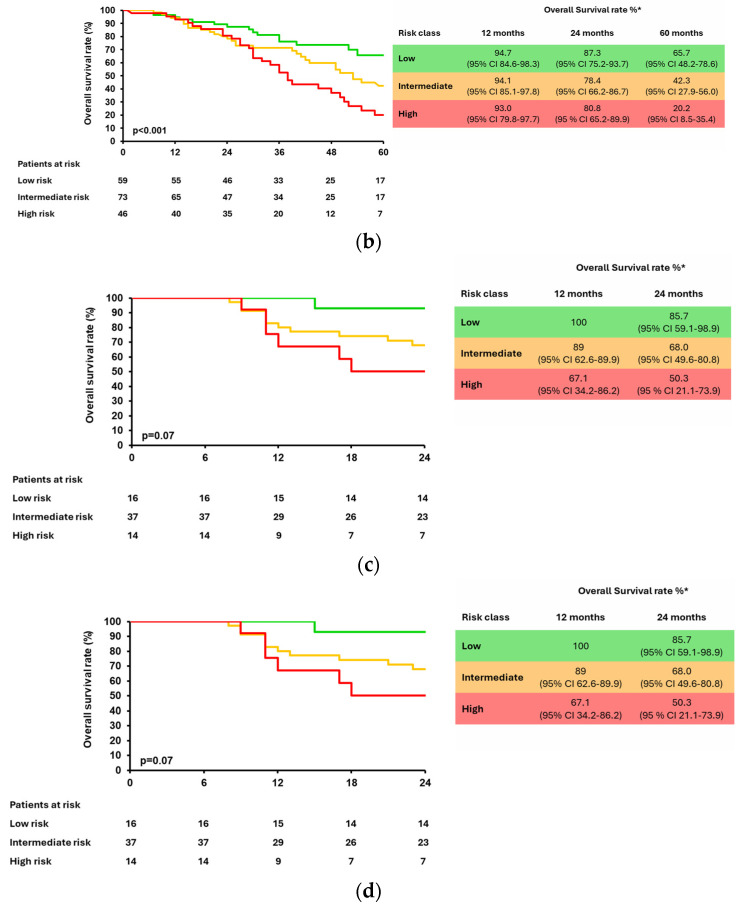
Overall 5-year survival rate stratified according to BCLC and mCCI class: (**a**) BCLC 0; (**b**) BCLC A; (**c**) BCLC B; (**d**) BCLC C. * Cumulative survival rate.

When stratified by BCLC stage, mCCI classes maintained their prognostic relevance in early-stage HCC. In BCLC 0 (N = 103) and A (N = 178), OS was significantly shorter in mCCI “high-“ and “intermediate-“ risk classes as compared to “low-risk” [BCLC 0, *p* = 0.02; BCLC A, *p* < 0.001] (Figure 5a,b). Differently, no significant differences among risk classes by mCCI in OS were observed among BCLC B (N = 67) and C (N = 41) patients (Figure 5c,d).

### 3.5. Post-Treatment Survival According to mCCI in Different BCLC Stages

A subanalysis was conducted to evaluate the association between comorbidity burden, expressed as mCCI risk class, and post-treatment survival. Given the limited sample size in specific treatment categories, the analyses were restricted to those most representative (Supplementary Table S2). Among patients with BCLC 0 treated with TA (N = 59), post-treatment survival did not differ significantly across the mCCI risk classes (*p* = 0.35), irrespective of liver function or radiological response. In contrast, among patients with BCLC A HCC (N = 68), the “high-risk” mCCI group was associated with significantly shorter post-treatment survival. This association was confirmed when the analysis was limited to CPT A patients with complete radiological response (N = 35), to minimize confounding by liver function and treatment outcome. In this subgroup, post-treatment survival was shorter in patients belonging in “high-risk” mCCI class as compared to those in “low/intermediate” classes, both after liver resection (median survival 53 [IQR 38–58] vs. 76 months [IQR 53–122], *p* = 0.03) and TA (median survival: 38 [IQR 30–83] vs. 80 [IQR 63–120] months, *p* = 0.03). These differences were not explained by disparities in HCC recurrence or access to subsequent lines of treatment which were comparable across mCCI risk groups.

Among patients with BCLC B HCC undergoing intra-arterial treatments (N = 34), survival curves for “intermediate” and “high-risk” mCCI classes substantially overlapped and were therefore grouped together and compared with the “low-risk” mCCI group. After stratification according to liver function and treatment response, a trend toward a longer post-treatment survival was observed in the “low-risk” group (median survival 57 [IQR 42–63] vs. 32 [IQR 12–37] months, *p* = 0.17), although this difference did not reach statistical significance (*p* = 0.17), regardless of access to further treatment lines. Finally, among patients with BCLC C undergoing systemic therapy (N = 24), post-treatment survival did not differ across the three mCCI risk classes (*p* = 0.05).

### 3.6. Exploratory Analysis of Comorbidity-Specific Survival Impact

An exploratory analysis was performed in 326 patients with complete comorbidity data to assess the association of individual comorbidities to OS. Baseline characteristics did not differ between patients who were included in the subanalysis and those excluded, except for mCCI classes who showed a lower overall comorbidity burden in the 326 included patients. Comorbidities were grouped as follows: cardiovascular diseases (any etiology), neurological diseases (including transient ischemic attacks and cerebrovascular accidents, hemiplegia, dementia), diabetes mellitus, moderate to severe chronic kidney disease (defined as eGFR <45 mL/min/1.73 m^2^), malignancy other than HCC (previous or current), chronic pulmonary disease, other comorbidities (at least one among peptic ulcer, peripheral vasculopathy, connective tissue disease). At univariable Cox regression analysis, older age was significantly associated with mortality (HR 1.03, 95% CI 1.01–1.05 *p* < 0.001), while diabetes and cardiovascular disease showed trends toward shorter survival (HR 1.32 95% CI 0.97–1.81, *p* = 0.08; HR 1.37, 95% CI 0.95–1.97, *p* = 0.09, respectively). In a multivariable analysis adjusting for tumor burden and AFP levels, older age (HR 1.04, 95% CI 1.02–1.06, *p* < 0.001) and cardiovascular disease (HR1.57, 95% CI 1.02–2.42, *p* = 0.04) remained independently associated with mortality (Supplementary Table S3).

## 4. Discussion

In a landscape characterized by an increasing number of therapeutic options for HCC at any stage, as well as an increasing median age of patients, there is still an unmet need for simple tools to quantify comorbidities and frailty to support longitudinal prognostic stratification and multidisciplinary evaluation of patients. This study pioneers the investigation of a modified CCI in HCC patients across all disease stages and treatment modalities. We demonstrated that by excluding points related to liver disease and HCC, the mCCI offers superior patient stratification compared to the original CCI. This enhanced stratification was associated with a distribution of treatment strategies among patients in intermediate stages, revealing a lower proportion of curative therapies in patients with higher comorbidity burden. The mCCI emerged as an independent predictor of intermediate-term mortality, alongside AFP, cancer stage, and liver function. Subanalyses further revealed that higher mCCI risk was associated with reduced survival in compensated cirrhosis patients undergoing curative resection or ablation for early HCC. Similarly, BCLC B patients with higher mCCI classes treated with TACE experienced greater post-treatment mortality. An exploratory analysis highlighted age and cardiovascular diseases as key drivers of survival impact within the CCI framework.

Prior studies on CCI in patients with HCC predominantly focused on early-stage, curative treatment candidates, often excluding age and HCC points from the score [16,17,18,19]. Our study, in contrast, encompassed all HCC stages and treatments, retaining age in the mCCI calculation and excluding both points coming from HCC and underlying liver disease. This comprehensive approach, aligned with multiparametric assessment [5], integrating comorbidities with tumor burden, liver function, and ECOG-PS to evaluate their collective impact on survival and treatment allocation. In the original CCI, the classifications of both liver disease and solid tumors failed to capture all the nuances that can significantly influence the therapeutic strategy chosen for a specific patient. Firstly, Charlson et al. grouped together chronic hepatitis and cirrhosis without portal hypertension as “mild” liver disease [6], even though the risk of decompensating events, HCC recurrence and prognosis can differ significantly between these groups. Furthermore, CCI broad classification of solid tumors as “localized” or “metastatic” [6] overlooks the wide variations in HCC prognosis and management driven by intrahepatic tumor burden and macrovascular invasion [21,22,23,24,25,28,29]. To avoid artificial score inflation and better reflect the true impact of comorbidities, we modified the CCI by excluding points related to HCC and underlying liver disease. This mCCI significantly improved patient stratification and was associated with treatment allocation patterns and overall survival. Critically, among patients with higher mCCI scores in intermediate stages, a lower proportion of curative treatments, particularly surgical options (50% vs. 72%), was observed. These observations suggest that the mCCI may capture the burden of comorbidities, serving as a useful tool for multidisciplinary patient evaluation [6]. However, given the observational design of the study and the multiple subgroup comparisons performed, these associations should be interpreted cautiously and not as causal effects on treatment allocation.

While a higher CCI is a known predictor of shorter OS in other cancers and some early HCC studies [11,12,13,14], the mCCI has been explored predominantly in liver transplantation, where it correlated with detrimental 90-day survival and OS [8]. Our study extends these findings by demonstrating that intermediate or high mCCI risk classes predict shorter OS across the entire HCC cohort. Survival curves revealed distinct separations at 18 months (“high-risk” vs. “low-intermediate risk”) and 30 months (“intermediate” vs. “low-risk”), indicating a significant, intermediate-to-long term impact of comorbidities on survival. This aligns with the predictive capacity of the original CCI in other contexts and the natural history of treated HCC [6,25]. Early mortality in the overall HCC cohort was primarily driven by tumor burden and liver function (BCLC C: median OS 17 months; BCLC D: 14 months). The observed shorter OS in intermediate- and high-risk mCCI classes within very early/early HCC further substantiates our hypothesis. A multivariable model, incorporating BCLC stage, AFP, CPT, and mCCI, performed well, confirming the complex interplay between liver function, tumor burden, biology, and comorbidities. Importantly, mCCI retained its prognostic significance after adjustment for established tumor- and liver-related variables, supporting its role as a complementary dimension of risk assessment rather than a surrogate of existing scores.

The apparent discrepancy between the association of mCCI and OS and the modest overall differences in first-line treatment allocation across mCCI classes can be related to the observed intermediate-to-long-term impact of the comorbidity burden in HCC patients. While initial treatment allocation is largely driven by tumor burden and liver function according to BCLC recommendations, the burden of extrahepatic comorbidities may exert a stronger influence on long-term outcomes through competing mortality risks, treatment tolerance, and the feasibility of subsequent therapeutic lines. Therefore, comorbidity burden may have a limited impact on the initial treatment decision but a more substantial influence on intermediate- and long-term survival.

Previous CCI studies primarily explored the association of comorbidity burden to shorter survival in the setting of liver resection [17,18,19], overlooking the impact on patients undergoing non-surgical treatment. To address this gap, our research investigated mCCI’s relationship with post-treatment survival across diverse therapeutic strategies. Patients with BCLC A HCC and higher mCCI exhibited shorter survival following both resection and thermal ablation. These outcomes appeared independent of HCC recurrence or subsequent treatment lines, suggesting a link to the patient’s overall health status rather than solely cancer or liver function. For BCLC B patients with preserved liver function but high/intermediate mCCI who achieved complete or partial response to TACE, a trend towards shorter OS was observed compared to those with lower comorbidity burden, irrespectively of access to second line treatments. These findings highlight that comorbidities not only limit the access to curative treatment in intermediate stage but can also influence outcomes in patients treated with intra-arterial treatment, necessitating careful consideration in multidisciplinary discussions. We acknowledge that our cohort differs from Charlson’s original population, and the prognostic weight of the individual comorbidities composing the score may vary in HCC patients. An exploratory analysis, despite limitations imposed by small sample size preventing granular stratification and possible selection bias due to the exclusion of patients without information about the single comorbidities composing the CCI score, revealed that age and cardiovascular disease independently predicted mortality in our population. These findings align with prior research and are attributable to reduced access to invasive treatments and increased complication risks in older patients or those with cardiovascular conditions [38,39,40]. Moreover, cardiovascular disease is a recognized cause of non-liver-related death in early HCC [41].

From a clinical perspective, the association we observed between comorbidities and survival underlines how a systematic assessment of extrahepatic comorbidity burden may complement existing prognostic frameworks used in multidisciplinary tumor boards. In practice, mCCI could be evaluated alongside BCLC stage and liver function to help identify patients in whom additional evaluation of frailty or cardiometabolic risk may be warranted before selecting invasive or curative strategies. Such an approach may support a more structured assessment of “fit” versus “unfit” patients within the multidisciplinary decision-making paradigm.

Our study’s single-center, retrospective design represents a limitation, introducing potential unmeasured confounding factors (frailty measures, sarcopenia, or socioeconomic variables not systematically captured in the retrospective dataset) that limit causal inference. In addition, the retrospective assessment of comorbidities from clinical records may have resulted in under-reporting of less-severe conditions and non-differential misclassification. The long study period (2011–2022) could represent another limit of our study, as therapeutic strategies (including criteria of access to LT and systemic therapies for advanced HCC) radically changed along these years. To explore this potential temporal effect, we stratified patients according to the year of HCC diagnosis, and the prognostic association between mCCI and survival remained consistent across the two cohorts. We also acknowledge that the monocentric nature of the study may limit the generalizability of the findings, as it reflects the clinical practice approach of a single Italian Center. To overcome these limitations, multi-center, prospective studies including mCCI systematically in multidisciplinary evaluation and treatment allocation are needed. Such studies would allow a more comprehensive assessment of mCCI’s impact at baseline and over time, including its relationship with treatment allocation and on liver- and non-liver-related mortality.

Moreover, future research should focus on refining mCCI or developing new scores that comprehensively integrate HCC severity and chronic liver disease burden and frailty. Such models would offer more robust tools for optimizing HCC patient management. Furthermore, investigating mCCI’s interactions with tumor-specific biomarkers and patient frailty indices could enhance risk stratification, facilitating personalized and effective treatment strategies and leading to improved patient outcomes.

## 5. Conclusions

In conclusion, our study underscores the importance of incorporating comorbidity burden into the evaluation of patients with HCC. The mCCI was associated with survival and allowed improved stratification of comorbidity burden compared with the standard CCI, supporting its potential role in prognostic assessment and multidisciplinary discussions alongside tumor stage and liver function.

These findings advocate for a multiparametric decision-making framework that extends beyond tumor and liver disease characteristics. Further prospective studies and external validation are warranted to confirm the clinical utility of mCCI in different HCC populations.

## Figures and Tables

**Table 1:** Comorbidities included in Charlson Comorbidity Index and point system.

Variable	Definition	Points
Age, years		1 for each decade >50
Myocardial infarction (MI)	History of definite or probable MI (Electrocardiographic signs of ischemia or elevation of cardiac enzymes)	1
Congestive heart failure	Exertional or paroxysmal nocturnal dyspnea and has responded to digitalis, diuretics, or afterload reducing agents	1
Peripheral vascular disease	Intermittent claudication; past bypass for chronic arterial insufficiency; history of gangrene/acute arterial insufficiency; untreated thoracic or abdominal aneurysm (≥6 cm)	1
Cerebrovascular accident or transient ischemic attack		1
Hemiplegia		2
Dementia	Chronic cognitive impairment	1
Chronic pulmonary disease		1
Connective tissue disease		1
Peptic ulcer disease	Ulcer disease with or without bleeding	1
Liver disease	Mild: chronic hepatitis/cirrhosis without portal hypertension	1
	Moderate/Severe: cirrhosis with portal hypertension	3
Diabetes	Uncomplicated, on treatment	1
	End-stage complications	2
Moderate to severe chronic kidney disease	Moderate: creatinine >3 mg/dL Severe: dialysis, kidney transplant, uremia	2
Solid tumor	Localized	2
	Metastatic	6
Leukemia		2
Lymphoma		2
AIDS		6

**Table 2:** Clinical and demographic characteristics of 401 patients included in the study.

Variables	Patients N = 401
Age, years *	68 (60–75)
Males, N (%)	298 (74)
Etiology, N (%)	
HCV	222 (55)
HBV	27 (7)
HDV	7 (2)
HBV + HCV	6 (1)
Non-viral	101 (25)
Mixed	38 (10)
Cirrhosis, N (%)	357 (89)
Ascites, N (%)	87 (22)
Encephalopathy, N (%)	27 (7)
Esophagogastric varices, N (%)	103 (29) ˣ
Albumin, g/dL *	4.00 (3.6–4.4)
Total bilirubin, mg/dL *	0.9 (0.7–1.4)
PT-INR *	1.12 (1.04–1.23)
Creatinine, mg/dL *	0.86 (0.74–1.05)
Platelets, 10^9^/mL *	131 (85–184)
AFP, ng/mL *	10.8 (4.1–53.9)
AFP >200 ng/mL, N (%)	42 (12) ^†^
Child–Pugh Class, N (%)	
A	309 (80) ^‡^
B	72 (18) ^‡^
C	7 (2) ^‡^
MELD *	9 (8–11)
ALBI grade, N (%)	
1	189 (50) ^§^
2	175 (47) ^§^
3	11 (3) ^§^
BCLC stage, N (%)	
0	103 (26)
A	178 (44)
B	67 (17)
C	41 (10)
D	12 (3) ^
Nodules, N (%)	
1	254 (63)
2–3	97 (24)
>3	50 (13)
Largest nodule’s diameter, cm *	2.5 (1.7–4.1)
Presence of MVI, EHS, or both, N (%)	
MVI	19 (5)
EHS	6 (2)
Both	3 (1)
“Milan Criteria” out, N (%)	128 (32)
ECOG PS, N (%)	
0	387 (97)
≥1	14 (3)

Data are expressed as number (percentage), unless otherwise specified. * Median (IQR); ˣ data available in 355 patients; † data available in 336 patients; ‡ data available in 388 patients; § data available in 375 patients; ^ according to BCLC recommendations 1/12 BCLC D patients undergoing LT were reclassified as BCLC A in the analysis. HCV: hepatitis C; HBV: hepatitis B; HDV: hepatitis delta; PT-INR: prothrombin time international normalized ratio; AFP: alpha-fetoprotein; MELD: model for end-stage liver disease; ALBI score: albumin-bilirubin score; BCLC: Barcelona Clinic Liver Cancer; MVI, macrovascular invasion; EHS, extrahepatic spread; ECOG PS: performance status by Eastern Cooperative Oncology Group.

**Table 3:** Predictors of mortality any time during follow-up at univariable and multivariable analysis in the 401 patients included in the study.

		Univariable Analysis			Multivariable Analysis		
Variable	Variable Type	HR	95% CI	*p*-Value	HR	95% CI	*p*-Value
Age, years	Continuous	1.03	1.02–1.05	<0.001	Excluded for collinearity		
Born male	Yes vs. No	1.15	0.85–1.56	0.36			
DMT2	Yes vs. No	1.27	0.96–1.67	0.09			
mCCI_class	Categorical						
Low-risk		1 (base)			1 (base)		
Intermediate-risk		1.73	1.24–2.40	0.001	1.69	1.16–2.47	0.007
High-risk		2.23	1.54–3.23	<0.001	2.80	1.81–4.33	<0.001
Non-viral etiology	Yes vs. No	1.53	1.14–2.07	0.005	1.13	0.77–1.66	0.50
Cirrhosis	Yes vs. No	1.12	0.71–1.78	0.62			
Encephalopathy	Yes vs. No	1.90	1.20–3.10	0.006	Excluded for collinearity		
Ascites	Yes vs. No	1.27	0.93–1.73	0.13			
Varices	Yes vs. No	1.33	0.99–1.78	0.06			
Albumin, g/dL	Continuous	0.71	0.56–0.89	0.004	Excluded for collinearity		
Bilirubin, mg/dL	Continuous	1.10	0.97–1.25	0.13			
Creatinine, mg/dL	Continuous	1.24	0.78–1.98	0.36			
Platelets <150,000/uL	Yes vs. No	0.97	0.73–1.29	0.83			
AFP > 200 ng/mL	Yes vs. No	1.92	1.31–2.81	0.001	1.53	1.02–2.30	0.04
Child–Pugh class	Categorical						0.03
A		1 (base)			1.50	1.04–2.16	
B/C		1.73	1.26–2.38	<0.001			
MELD	Continuous	1.06	1.01–1.10	0.01	Excluded for collinearity		
ALBI class	Categorical				Excluded for collinearity		
1		1 (base)					
2		1.29	0.98–1.71	0.07			
3		2.53	1.27–5.02	0.008			
Number of HCC nodules	Categorical				Excluded for collinearity		
1		1 (base)					
2–3		1.87	1.39–2.53	<0.001			
>3		3.15	2.15–4.62	<0.001			
Maximum HCC diameter, cm	Continuous	1.01	1.01–1.02	<0.001	Excluded for collinearity		
MVI/EHS	Categorical				Excluded for collinearity		
No		1 (base)					
MVI		5.27	3.23–8.61	<0.001			
EHS		2.00	0.74–5.43	0.17			
Both		13.19	4.10–42.41	<0.001			
BCLC	Categorical						
0		1 (base)			1 (base)		
A		1.62	1.14–2.30	0.007	1.58	1.06–2.37	0.03
B		2.75	1.81–4.18	<0.001	3.43	2.13–5.43	<0.001
C		6.93	4.39–10.93	<0.001	8.86	5.08–15.5	<0.001
D		10.37	5.29–20.31	<0.001	9.03	4.07–20.0	<0.001

mCCI: modified Charlson Comorbidity Index; AFP: alpha-fetoprotein; MELD: model for end-stage liver disease; ALBI score: albumin-bilirubin score; BCLC: Barcelona Clinic Liver Cancer; MVI, macrovascular invasion; EHS, extrahepatic spread.

## Data Availability

Data, analytical methodologies, and research materials will be accessible to other investigators upon request to the corresponding author.

## Supplementary Materials

The following supporting information can be downloaded at: https://www.mdpi.com/article/10.3390/cancers18071151/s1, Figure S1: Discrimination and calibration of mCCI for prediction of survival.; Figure S2: Cumulative hazard plots stratified by mCCI risk classes; Figure S3: Discrimination and calibration of the multivariable model for prediction of survival; Table S1: Distribution of mCCI risk classes across BCLC stages.; Table S2: First-line treatment allocation in 401 newly diagnosed HCC according to BCLC stage and mCCI class; Table S3: Predictors of mortality any time during follow-up at univariable and multivariable analysis in the 326 patients with available information on the comorbidities composing CCI.

## Abbreviations

The following abbreviations are used in this manuscript:

AFP	Alpha-Fetoprotein
ALBI	Albumin-Bilirubin
ALD	Alcoholic Liver Disease
BCLC	Barcelona Clinic Liver Cancer
BSC	Best Supportive Care
CCI	Charlson Comorbidity Index
CI	Confidence Interval
CPT	Child–Pugh–Turcotte
CT	Computed Tomography
ECOG PS	Eastern Cooperative Oncology Group Performance Status
EHS	Extrahepatic Spread
HBV	Hepatitis B Virus
HCC	Hepatocellular Carcinoma
HCV	Hepatitis C Virus
HDV	Hepatitis Delta Virus
HR	Hazard Ratio
INR	International Normalized Ratio
IQR	Interquartile Range
LT	Liver Transplantation
LRT	Locoregional Treatment
MASLD	Metabolic-Associated Steatotic Liver Disease
mCCI	Modified Charlson Comorbidity Index
MELD	Model for End-Stage Liver Disease
MR	Magnetic Resonance
MVI	Macrovascular Invasion
OS	Overall Survival
TA	Thermal Ablation
TACE	Transarterial Chemoembolization
TARE	Transarterial Radioembolization
UEG	Upper Gastrointestinal Endoscopy

## References

[1] Sung H., Ferlay J., Siegel R.L., Laversanne M., Soerjomataram I., Jemal A., Bray F. (2021). Global Cancer Statistics 2020: GLOBOCAN Estimates of Incidence and Mortality Worldwide for 36 Cancers in 185 Countries. CA Cancer J. Clin..

[2] Toh M.R., Wong E.Y.T., Wong S.H., Ng A.W.T., Loo L.-H., Chow P.K.-H., Ngeow J. (2023). Global Epidemiology and Genetics of Hepatocellular Carcinoma. Gastroenterology.

[3] Forner A., Reig M., Bruix J. (2018). Hepatocellular carcinoma. Lancet.

[4] Reig M., Forner A., Rimola J., Ferrer-Fàbrega J., Burrel M., Garcia-Criado Á., Kelley R.K., Galle P.R., Mazzaferro V., Salem R. (2021). BCLC strategy for prognosis prediction and treatment recommendation: The 2022 update. J. Hepatol..

[5] Vitale A., Cabibbo G., Iavarone M., Viganò L., Pinato D.J., Ponziani F.R., Lai Q., Casadei-Gardini A., Celsa C., Galati G. (2023). Personalised management of patients with hepatocellular carcinoma: A multiparametric therapeutic hierarchy concept. Lancet Oncol..

[6] Charlson M.E., Pompei P., Ales K.L., MacKenzie C.R. (1987). A new method of classifying prognostic comorbidity in longitudinal studies: Development and validation. J. Chronic Dis..

[7] Fleming K.M., Aithal G.P., Card T.R., West J. (2011). All-cause mortality in people with cirrhosis compared with the general population: A population-based cohort study. Liver Int..

[8] Coppel S., Mathur K., Ekser B., Patidar K.R., Orman E., Desai A.P., Vilar-Gomez E., Kubal C., Chalasani N., Nephew L. (2020). Extra-hepatic comorbidity burden significantly increases 90-day mortality in patients with cirrhosis and high model for endstage liver disease. BMC Gastroenterol..

[9] Jepsen P., Vilstrup H., Andersen P., Lash T., Sorensen H. (2008). Comorbidity and survival of Danish cirrhosis patients: A nationwide population-based cohort study. J. Hepatol..

[10] Artru F., Sacleux S.-C., Ursic-Bedoya J., Wandji L.C.N., Lutu A., L’Hermite S., Levy C., Khaldi M., Levesque E., Dharancy S. (2025). Long-term outcome following liver transplantation of patients with ACLF grade 3. J. Hepatol..

[11] Mariotto A.B., Wang Z., Klabunde C.N., Cho H., Das B., Feuer E.J. (2013). Life tables adjusted for comorbidity more accurately estimate noncancer survival for recently diagnosed cancer patients. J. Clin. Epidemiol..

[12] Marventano S., Grosso G., Mistretta A., Bogusz-Czerniewicz M., Ferranti R., Nolfo F., Giorgianni G., Rametta S., Drago F., Basile F. (2014). Evaluation of four comorbidity indices and Charlson comorbidity index adjustment for colorectal cancer patients. Int. J. Color. Dis..

[13] Kjær E.K.R., Jensen J.S., Jakobsen K.K., Lelkaitis G., Wessel I., von Buchwald C., Grønhøj C. (2021). The Impact of Comorbidity on Survival in Patients with Head and Neck Squamous Cell Carcinoma: A Nationwide Case-Control Study Spanning 35 Years. Front. Oncol..

[14] Dhakal P., Lyden E., Joshi U., Pyakuryal A., Loh K.P., Klepin H., Bhatt V.R. (2023). Comorbidity burden and outcomes of older adults with acute promyelocytic leukemia: A National Cancer Database analysis of 2221 patients. Leuk. Lymphoma.

[15] Koseki Y., Hikage M., Fujiya K., Kamiya S., Tanizawa Y., Bando E., Terashima M. (2021). Utility of a modified age-adjusted Charlson Comorbidity Index in predicting cause-specific survival among patients with gastric cancer. Eur. J. Surg. Oncol. (EJSO).

[16] Kanneganti M., Al-Hasan M., Bourque S., Deodhar S., Yang J.D., Huang D.Q., Kulkarni A.V., Gopal P., Parikh N.D., Kanwal F. (2024). Older Age but Not Comorbidity Is Associated with Worse Survival in Patients with Hepatocellular Carcinoma. Clin. Gastroenterol. Hepatol..

[17] Khandoga A., Drefs M., Schoenberg M., Schiergens T., Frenes K., Winkel M.O.D., Trumm C., Angele M.K., Guba M., Werner J. (2017). Differential significance of early surgical complications for acute and long-term recurrence-free survival following surgical resection of hepatocellular carcinoma: Do comorbidities play a role?. Eur. J. Gastroenterol. Hepatol..

[18] Eilard M.S., Helmersson M., Rizell M., Vaz J., Åberg F., Taflin H. (2025). Non-liver comorbidity in patients with hepatocellular carcinoma and curative treatments—A Swedish national registry study. Scand. J. Gastroenterol..

[19] Shinkawa H., Tanaka S., Takemura S., Amano R., Kimura K., Nishioka T., Miyazaki T., Kubo S. (2020). Predictive Value of the Age-Adjusted Charlson Comorbidity Index for Outcomes After Hepatic Resection of Hepatocellular Carcinoma. World J. Surg..

[20] Von Elm E., Altman D.G., Egger M., Pocock S.J., Gøtzsche P.C., Vandenbroucke J.P., Initiative S. (2007). The Strengthening the Reporting of Observational Studies in Epidemiology (STROBE) statement: Guidelines for reporting observational studies. Lancet.

[21] Bruix J., Sherman M. (2005). Practice Guidelines Committee, American Association for the Study of Liver Diseases Management of Hepatocellular Carcinoma *. Hepatology.

[22] Bruix J., Sherman M. (2011). Management of hepatocellular carcinoma: An update. Hepatology.

[23] European Association for the Study of the Liver (2012). European Organization for Research and Treatment of Cancer. EASL–EORTC clinical practice guidelines: Management of hepatocellular carcinoma. J. Hepatol..

[24] Heimbach J.K., Kulik L.M., Finn R.S., Sirlin C.B., Abecassis M.M., Roberts L.R., Zhu A.X., Murad M.H., Marrero J.A. (2018). AASLD guidelines for the treatment of hepatocellular carcinoma. Hepatology.

[25] European Association for the Study of the Liver (2018). EASL Clinical Practice Guidelines: Management of hepatocellular carcinoma. J. Hepatol..

[26] Pugh R.N.H., Murray-Lyon I.M., Dawson J.L., Pietroni M.C., Williams R. (1973). Transection of the oesophagus for bleeding oesophageal varices. Br. J. Surg..

[27] Oken M.M., Creech R.H., Tormey D.C., Horton J., Davis T.E., McFadden E.T., Carbone P.P. (1982). Toxicity and response criteria of the Eastern Cooperative Oncology Group. Am. J. Clin. Oncol..

[28] Llovet J.M., Brú C., Bruix J. (1999). Prognosis of hepatocellular carcinoma: The BCLC staging classification. Seminars in Liver Disease.

[29] Forner A., Reig M., de Lope C.R., Bruix J. (2010). Current Strategy for Staging and Treatment: The BCLC Update and Future Prospects. Semin. Liver Dis..

[30] De A., Bhagat N., Mehta M., Taneja S., Duseja A. (2024). Metabolic dysfunction-associated steatotic liver disease (MASLD) definition is better than MAFLD criteria for lean patients with NAFLD. J. Hepatol..

[31] (2015). European Association for the Study of the Liver EASL Clinical Practice Guidelines: Autoimmune hepatitis. J. Hepatol..

[32] Hirschfield G.M., Beuers U., Corpechot C., Invernizzi P., Jones D., Marzioni M., Schramm C. (2017). EASL Clinical Practice Guidelines: The diagnosis and management of patients with primary biliary cholangitis. J. Hepatol..

[33] Bedossa P., Poynard T. (1996). An Algorithm for the Grading of Activity in Chronic Hepatitis C. Hepatology.

[34] Kamath P.S., Wiesner R.H., Malinchoc M., Kremers W., Therneau T.M., Kosberg C.L., D’Amico G., Dickson R.E., Kim R.W. (2001). A Model to Predict Survival in Patients with End–Stage Liver Disease. Hepatology.

[35] Johnson P.J., Berhane S., Kagebayashi C., Satomura S., Teng M., Reeves H.L., O’Beirne J., Fox R., Skowronska A., Palmer D. (2015). Assessment of Liver Function in Patients with Hepatocellular Carcinoma: A New Evidence-Based Approach—The ALBI Grade. J. Clin. Oncol..

[36] de Franchis R., Bosch J., Garcia-Tsao G., Reiberger T., Ripoll C., Abraldes J.G., Albillos A., Baiges A., Bajaj J., Bañares R. (2022). Baveno VII–Renewing consensus in portal hypertension. J. Hepatol..

[37] Arrieta O., Cacho B., Morales-Espinosa D., Ruelas-Villavicencio A., Flores-Estrada D., Hernández-Pedro N. (2007). The progressive elevation of alpha fetoprotein for the diagnosis of hepatocellular carcinoma in patients with liver cirrhosis. BMC Cancer.

[38] Tzeng C.-W.D., Cooper A.B., Vauthey J.-N., Curley S.A., Aloia T.A. (2014). Predictors of morbidity and mortality after hepatectomy in elderly patients: Analysis of 7621 NSQIP patients. HPB.

[39] Longbotham D., Young A., Nana G., Feltbower R., Hidalgo E., Toogood G., Lodge P.A., Attia M., Prasad K.R. (2020). The impact of age on post-operative liver function following right hepatectomy: A retrospective, single centre experience. HPB.

[40] Ruzzenente A., Conci S., Ciangherotti A., Campagnaro T., Valdegamberi A., Bertuzzo F., Bagante F., Mantovani G., De Angelis M., Dorna A.E. (2017). Impact of age on short-term outcomes of liver surgery. Medicine.

[41] El Dahan K.S., Daher D., Rich N.E., Nayak A.J., Ankoma-Sey C., Govalan R., Bhongade M.B., Molina E., Amador E., Pitts H. (2025). Causes of mortality among patients with early-stage hepatocellular carcinoma. Hepatology.

