# Supplementary material for: Modified Charlson Comorbidity Index to Improve Management of Patients with Hepatocellular Carcinoma: A Step Towards Multiparametric Approach
Source: Cancers (Basel). 2026 Apr 2;18(7):1151. doi: 10.3390/cancers18071151 (PMC13072432; doi:10.3390/cancers18071151)
Supplement: Supplementary file 1 [file cancers-18-01151-s001.zip › cancers-4185624-supplementary-tables.pdf]

Table S1. Distribution of mCCI risk classes across BCLC stages

BCLC stage	Total	mCCI class Low risk N (%)	mCCI class Intermediate risk N (%)	mCCI class High risk N (%)
0	103	29 (28)	55 (54)	19 (18)
A	178	59 (33)	73 (41)	46 (26)
B	67	16 (24)	37 (55)	14 (21)
C	41	13 (32)	22 (54)	6 (14)

Table S2. First line treatment allocation in 401 newly diagnosed HCC according to BCLC stage and mCCI class.

Whole population					
**First-line treatments, N (%)**	**Overall (N=401)**	**mCCI class Low risk (N=124)**	**mCCI class Intermediate risk (N=191)**	**mCCI class High risk (N=86)**	**p-value**
					0.07
Liver Transplantation	24 (6)	14 (12)	9 (5)	1 (1)	
Resection	77 (19)	30 (24)	34 (18)	13 (15)	
Ablation	138 (35)	35 (28)	68 (35)	35 (41)	
TACE	112 (28)	30 (24)	54 (28)	28 (32)	
TARE	4 (1)	0	2 (1)	2 (2)	
Systemic treatment	33 (8)	11 (9)	17 (9)	5 (6)	
Supportive care only	13 (3)	4 (3)	7 (4)	3 (3)	
Treatments with curative intent*	239 (60)	79 (64)	111 (58)	49 (57)	0.52
Response to first-line treatment					0.81
Complete response	244 (61)	79 (64)	117 (61)	48 (56)	
Partial response	73 (18)	21 (17)	34 (18)	18 (21)	
Stable disease	14 (4)	4 (3)	5 (3)	5 (6)	
Progressive disease	70 (17)	20 (16)	35 (18)	15 (17)	
HCC recurrence any time after complete response	166 (68)	50 (63)	83 (71)	33 (69)	0.53
**BCLC 0**					
**First-line treatments, N (%)**	**Overall (N=103)**	**mCCI class Low risk (N=29)**	**mCCI class Intermediate risk (N=55)**	**mCCI class High risk (N=19)**	**p-value**
					0.27
Liver Transplantation	5 (5)	3 (10)	1 (5)	1 (1)	
Resection	15 (15)	6 (21)	8 (18)	1 (15)	
Ablation	59 (57)	12 (41)	33 (35)	14 (41)	
TACE	21 (20)	8 (28)	11 (28)	2 (32)	

TARE<br>Systemic treatment<br>Supportive care only	-<br>-<br>3 (3)	-<br>-<br>0	-<br>-<br>2 (4)	-<br>-<br>1 (3)	
Treatments with curative intent*	79 (77)	21 (71)	42 (58)	16 (57)	0.63
Response to first-line treatment<br>Complete response<br>Partial response<br>Stable disease<br>Progressive disease	 244 (61)<br>73 (18)<br>14 (4)<br>70 (17)	 79 (64)<br>21 (17)<br>4 (3)<br>20 (16)	 117 (61)<br>34 (18)<br>5 (3)<br>35 (18)	 48 (56)<br>18 (21)<br>5 (6)<br>15 (17)	0.81
HCC recurrence any time after complete response	60 (71)	15 (62)	35 (76)	10 (71)	0.49
BCLC A					
**First-line treatments, N (%)**	**Overall (N=178)**	**mCCI class Low risk (N=59)**	**mCCI class Intermediate risk (N=73)**	**mCCI class High risk (N=46)**	**p-value**
 Liver Transplantation<br>Resection<br>Ablation<br>TACE<br>TARE<br>Systemic treatment<br>Supportive care only	 11 (6)<br>44 (25)<br>68 (38)<br>50 (28)<br>-<br>3 (2)<br>2 (1)	 7 (12)<br>17 (29)<br>23 (39)<br>10 (17)<br>-<br>2 (3)<br>0	 4 (6)<br>17 (23)<br>25 (34)<br>25 (34)<br>-<br>0<br>2 (3)	 0<br>10 (22)<br>20 (43)<br>15 (33)<br>-<br>1 (2)<br>0	0.09
**Treatments with curative intent***	178 (69)	47 (80)	46 (63)	30 (65)	0.10
**Response to first-line treatment**<br>**Complete response**<br>**Partial response**<br>**Stable disease**<br>**Progressive disease**	 244 (61)<br>73 (18)<br>14 (4)<br>70 (17)	 79 (64)<br>21 (17)<br>4 (3)<br>20 (16)	 117 (61)<br>34 (18)<br>5 (3)<br>35 (18)	 48 (56)<br>18 (21)<br>5 (6)<br>15 (17)	0.81
**HCC recurrence any time after complete response**	120 (67)	52 (88)	40 (55)	28 (61)	0.46
**BCLC B**					
**First-line treatments, N (%)**	**Overall (N=67)**	**mCCI class Low risk (N=16)**	**mCCI class Intermediate risk (N=37)**	**mCCI class High risk (N=14)**	**p-value**
 Liver Transplantation<br>**Resection**<br>**Ablation**<br>**TACE**<br>**TARE**<br>**Systemic treatment**<br>**Supportive care only**	 5 (8)<br>14 (21)<br>8 (12)<br>31 (46)<br>3 (4)<br>6 (9)<br>-	 2 (12)<br>5 (24)<br>0 (28)<br>8 (24)<br>0<br>1 (9)<br>-	 3 (8)<br>8 (22)<br>7 (19)<br>13 (35)<br>2 (5)<br>4 (11)<br>-	 0<br>1 (7)<br>1 (7)<br>10 (72)<br>1 (7)<br>1 (7)<br>-	0.07

Treatments with curative intent*	239 (60)	79 (64)	111 (58)	12 (86)	0.08
**BCLC C**					
**First-line treatments, N (%)**	**Overall (N=41)**	**mCCI class Low risk (N=13)**	**mCCI class Intermediate risk (N=22)**	**mCCI class High risk (N=6)**	**p-value**
**Liver Transplantation**	2 (5)	1 (8)	1 (4)	0	
**Resection**	4 (10)	2 (15)	1 (4)	1 (16.6)	
**Ablation**	3 (7)	0	3 (14)	0	
**TACE**	5 (12)	1 (8)	3 (14)	1 (16.6)	
**TARE**	1 (2)	0	0	1 (16.6)	
**Systemic treatment**	24 (59)	8 (61)	13 (59)	3 (50)	
**Supportive care only**	2 (5)	1 (8)	1 (5)	0	
**Treatments with curative intent***	9 (22)	3 (23)	5 (22)	1 (16.6)	0.94

* Treatments with curative intent included: liver transplantation (LT), resection and radiofrequency/microwave thermal ablation (TA)
mCCI: modified Charlson Comorbidity Index; HCC: hepatocellular carcinoma; BCLC: Barcelona Clinic Liver Cancer; TACE: transarterial chemoembolization; TARE: transarterial radioembolization;

Table S3. Predictors of mortality any time during follow up at univariable and multivariable analysis in the 326 patients with available information on the comorbidities composing CCI

		**Univariable Analysis**			**Multivariable analysis**		
**Variable**	**Variable Type**	**HR**	**95% CI**	**p-value**	**HR**	**95% CI**	**p-value**
Age, years	Continuous	1.03	1.01-1.05	0.002	1.04	1.02-1.06	**<0.001**
Born male	Yes vs No	1.26	0.88-1.82	0.21			
DMT2	Yes vs No	1.32	0.97-1.81	0.08	1.14	0.75-1.71	0.50
Cardiovascular disease*	Yes vs No	1.37	0.95-1.97	0.09	1.57	1.02-2.42	**0.04**
Neurological disease[x]	Yes vs No	1.33	0.49-3.58	0.58			
Severe or moderate to severe kidney disease	Yes vs No	1.26	0.82-1.92	0.29			
Chronic pulmonary disease	Yes vs No	0.99	0.58-1.68	0.96			
Malignancies other than HCC	Yes vs No	1.00	0.59-1.69	0.99			
Other comorbidities†	Yes vs No	1.62	0.90-2.93	0.11			
Non viral etiology	Yes vs No	1.52	1.08-2.13	0.02	1.07	0.67-1.71	0.80
Cirrhosis	Yes vs No	1.06	0.65-1.72	0.83			
Encephalopathy	Yes vs No	2.07	1.22-3.54	0.007	Excluded for collinearity		
Ascites	Yes vs No	1.19	0.84-1.69	0.33			
Varices	Yes vs No	1.34	0.95-1.88	0.09			
Albumin, g/dL	Continuous	0.76	0.58-0.99	0.04	Excluded for collinearity		
Bilirubin, mg/dL	Continuous	1.10	0.96-1.27	0.17			
Platelets <150,000/uL	Yes vs No	0.99	0.72-1.37	0.99			
AFP>200 ng/mL	Yes vs No	1.99	1.28-3.12	0.002	1.75	1.08-2.82	**0.02**
Child Pugh class B/C	Yes vs No	1.73	1.19-2.52	0.004	1.50	0.95-2.37	0.08
ALBI grade 2/3	Yes vs No	1.21	0.89-1.64	0.20	Excluded for collinearity		
BCLC	Categorical						
0		1 (base)			1 (base)		
A		1.60	1.07-2.38	0.02	1.53	0.96-2.46	0.08
B		2.82	1.75-4.54	<0.001	3.49	2.03-6.01	**<0.001**
C		7.72	4.60-12.9	<0.001	8.56	4.63-15.8	**<0.001**
D		10.2	4.79-21.3	<0.001	9.06	3.81 –21.5	**<0.001**

* Myocardial infarction (history of definite or probable myocardial infarction, electrocardiographic signs of ischemia or elevation of cardiac enzymes), congestive heart failure, peripheral vascular disease; [x]Cerebrovascular accident or transient ischemic attack, hemiplegia, dementia; †connective tissue disease, peptic ulcer disease. No patients had leukemia, lymphoma or AIDS.
