# Supplementary material for: Modified Charlson Comorbidity Index to Improve Management of Patients with Hepatocellular Carcinoma: A Step Towards Multiparametric Approach
Source: Cancers (Basel). 2026 Apr 2;18(7):1151. doi: 10.3390/cancers18071151 (PMC13072432; doi:10.3390/cancers18071151)
Supplement: Supplementary file 1 [file cancers-18-01151-s001.zip › cancers-4185624_Supplementary figures.pptx]

## Slide 1
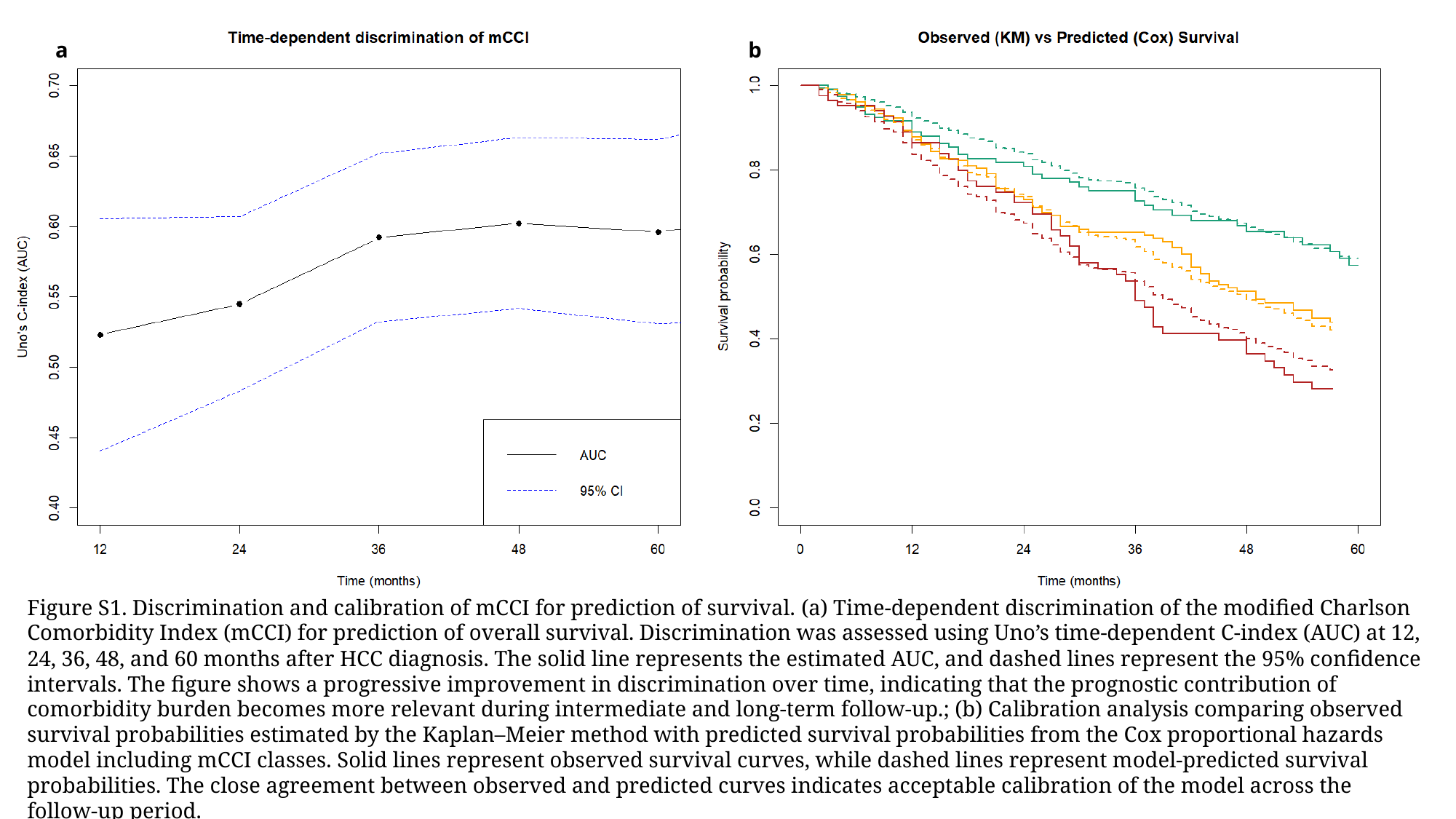

b
a
Figure S1. Discrimination and calibration of mCCI for prediction of survival. (a) Time-dependent discrimination of the modified Charlson Comorbidity Index (mCCI) for prediction of overall survival. Discrimination was assessed using Uno’s time-dependent C-index (AUC) at 12, 24, 36, 48, and 60 months after HCC diagnosis. The solid line represents the estimated AUC, and dashed lines represent the 95% confidence intervals. The figure shows a progressive improvement in discrimination over time, indicating that the prognostic contribution of comorbidity burden becomes more relevant during intermediate and long-term follow-up.; (b) Calibration analysis comparing observed survival probabilities estimated by the Kaplan–Meier method with predicted survival probabilities from the Cox proportional hazards model including mCCI classes. Solid lines represent observed survival curves, while dashed lines represent model-predicted survival probabilities. The close agreement between observed and predicted curves indicates acceptable calibration of the model across the follow-up period.

## Slide 2
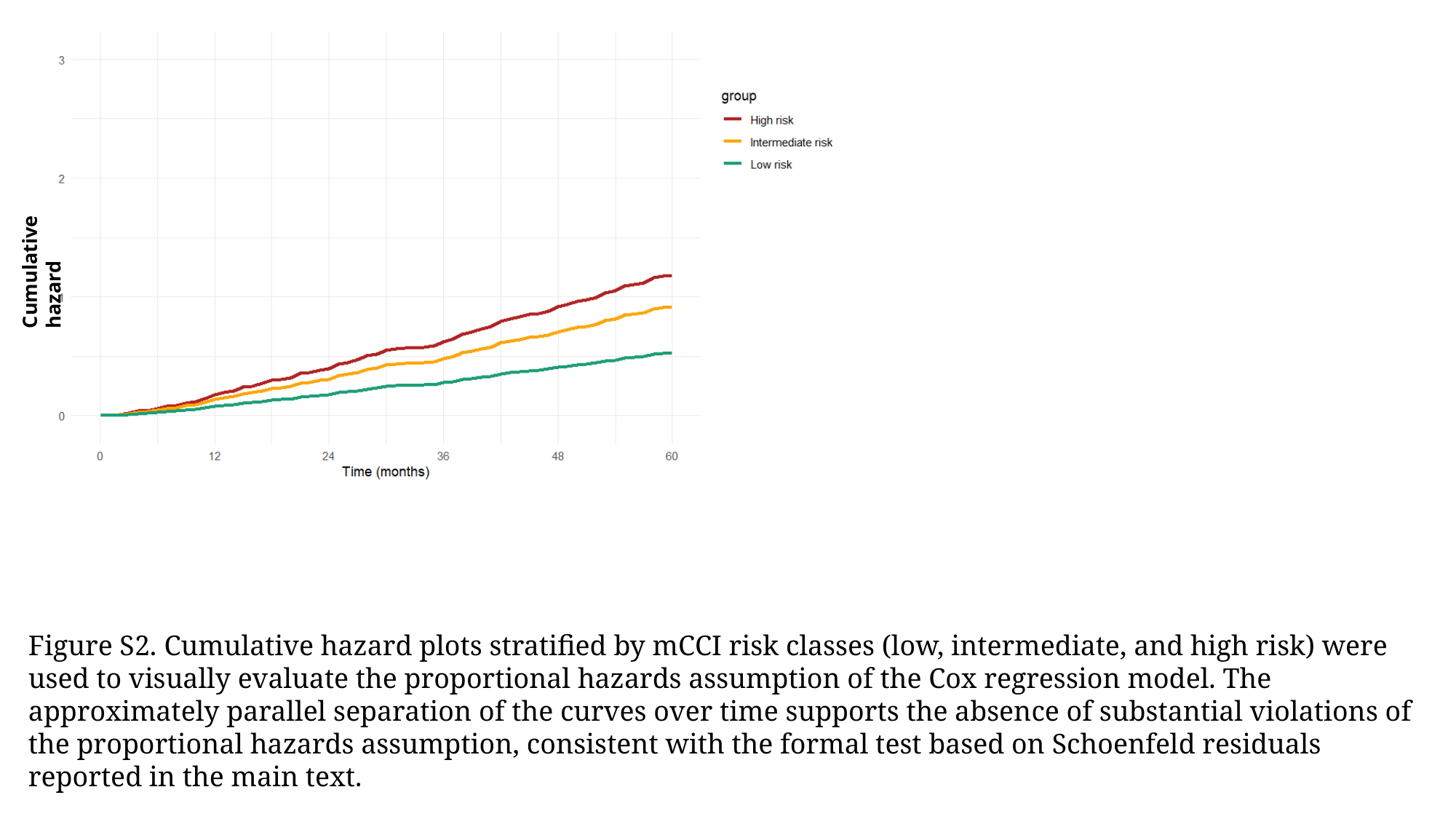

Cumulative hazard
Figure S2. Cumulative hazard plots stratified by mCCI risk classes (low, intermediate, and high risk) were used to visually evaluate the proportional hazards assumption of the Cox regression model. The approximately parallel separation of the curves over time supports the absence of substantial violations of the proportional hazards assumption, consistent with the formal test based on Schoenfeld residuals reported in the main text.

## Slide 3
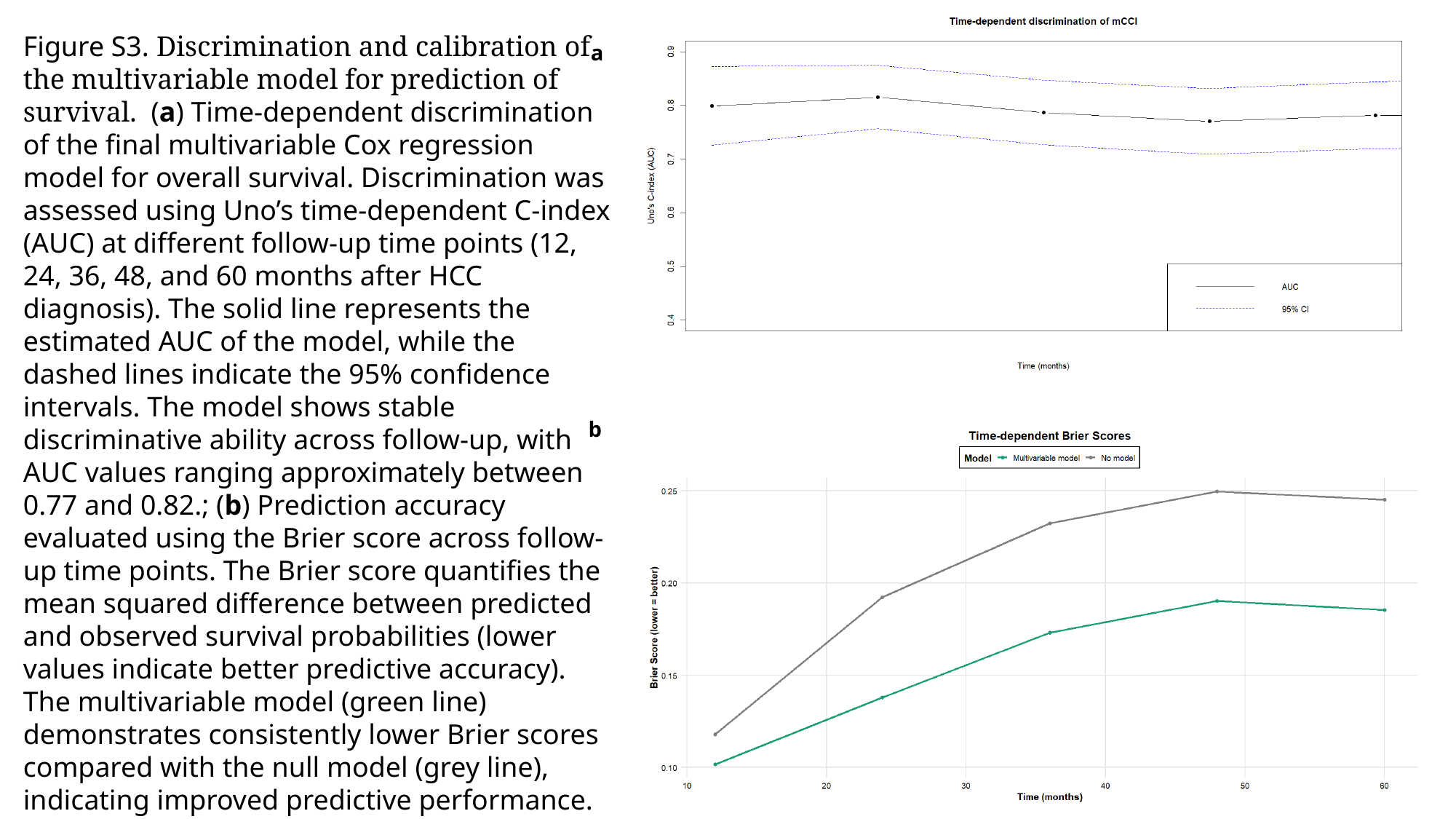

Figure S3. Discrimination and calibration of the multivariable model for prediction of survival. (a) Time-dependent discrimination of the final multivariable Cox regression model for overall survival. Discrimination was assessed using Uno’s time-dependent C-index (AUC) at different follow-up time points (12, 24, 36, 48, and 60 months after HCC diagnosis). The solid line represents the estimated AUC of the model, while the dashed lines indicate the 95% confidence intervals. The model shows stable discriminative ability across follow-up, with AUC values ranging approximately between 0.77 and 0.82.; (b) Prediction accuracy evaluated using the Brier score across follow-up time points. The Brier score quantifies the mean squared difference between predicted and observed survival probabilities (lower values indicate better predictive accuracy). The multivariable model (green line) demonstrates consistently lower Brier scores compared with the null model (grey line), indicating improved predictive performance. The multivariable model included mCCI risk class, BCLC stage, alpha-fetoprotein (AFP), and Child–Pugh–Turcotte class.
a
b
